# Time trend analysis of thyroid cancer surgery in China: single institutional database analysis of 15,000 patients

**DOI:** 10.1007/s12020-020-02230-7

**Published:** 2020-03-02

**Authors:** Chengqiu Sui, Nan Liang, Rui Du, Qiao He, Daqi Zhang, Fang Li, Yantao Fu, Gianlorenzo Dionigi, Hui Sun

**Affiliations:** 1 Division of Thyroid Surgery, China–Japan Union Hospital of Jilin University, Jilin Provincial Key Laboratory of Surgical Translational Medicine, Jilin Provincial Precision Medicine Laboratory of Molecular Biology and Translational Medicine on Differentiated Thyroid Carcinoma, Changchun, Jilin Province China; 2 Division for Endocrine and Minimally Invasive Surgery, Department of Human Pathology in Adulthood and Childhood “G. Barresi”, University Hospital “G. Martino”, The University of Messina, Via Consolare Valeria 1, 98125 Messina, Italy

**Keywords:** Thyroid cancer, Incidence, Surgery, Cancer registration, China, Guidelines

## Abstract

**Purpose:**

The institutional database of the Thyroid Surgery Division in China–Japan Union Hospital of Jilin University was queried to audit time trend patterns in thyroid cancer (TC) management between 2008 and 2017.

**Methods:**

Retrospective longitudinal analysis. Clinicopathological features and treatment strategies were analyzed. Frequencies and multivariate tests were used to detect correlations.

**Results:**

Clinical data were obtained from 15,000 TC patients (i.e., 71.3% of 21,044 operations). Papillary was the most common histological subtype (*n* = 14,916, 99%), and 76% were microcarcinomas. Stage I (95%) and low-risk patients (58%) were prevalent throughout the 10-year period. The trend for total thyroidectomy increased from 29.1% (2008–2012) to 67.9% (2013–2015), and then dropped to 48.6% (2016–2017). A total of 8827 (52%) patients received central lymph node dissection (CLND). The tendency for CLND increased from 15.7 to 86.4% during the 10-year period. While the trend of lateral lymph node dissection decreased from 71.3 to 13.3%. Radioactive iodine therapy was offered to 10% of patients (2008–2012), except for a low value (5.4%) in 2009, and then increased from 12.3% (2012) to 41.3% (2015), while decreased to 32.4% (2017).

**Conclusion:**

The surgical management of TC patients has undergone continuous changes over the past 10 years. The evolution from aggressive treatment to a more conservative approach has been constant. Our results suggest that the current surgical management approach for TC is adequate and in support of the published guidelines. Our findings warrant further investigation to determine the clinical implications of decision making for TC.

## Introduction

An increase in thyroid cancer (TC) incidence has been reported by studies conducted in the People’s Republic of China [1–19]. In 2013, China had an overall age-standardized TC incidence rate of 7.56/100,000, according to the Chinese National Cancer Registry, which ranked seventh in overall cancers [1–19]. Analysis showed that the most commonly diagnosed group are females in their 30s and 40s. The crude mortality of TC in China is 0.52/100,000. The incidence and mortality rates of TC are higher in females than in males and higher in urban areas than in rural areas. Eastern areas had the highest incidence, followed by central and western areas [1].

Endocrinologists and surgeons have consistently focused on improving treatment, perioperative management, and radioactive iodine (RAI) therapy to enhance the outcomes of TC in China [20–24]. TC management guidelines have been translated, updated, and transferred into Chinese clinical practice (Table 1) [20–24]. Today, TC, which includes mostly papillary thyroid cancer (PTC), is a treatable disease with a good prognosis [16–19]. The core of TC management includes removal of the tumor either by thyroidectomy or thyroid-conserving surgery (i.e., lobectomy) [19]. However, no research has reported TC surgical trends or the influential factors of these trends in China [1–19]. This retrospective study aimed to investigate the rates of different thyroidectomies and related factors in TC patients to determine whether they played a role in the modalities of surgery performed at Thyroid Surgery Division in China–Japan Union Hospital of Jilin University over a 10-year period (2008–2017).

**Table 1:** Domestic and foreign DTC management guidelines over the 10-year period

Institution	Features	Recommendations	Recommendation rating	References^a^
2009 ATA guidelines	MTD > 1 cm, there are contralateral thyroid nodules, or regional or distant metastases, with personal history of radiation therapy to the head and neck, or first-degree family history of DTC, older age (>45 years)	Near-total or total thyroidectomy	A	112, 116, 122, 123, 156, 157, 161, 162
	Small (<1 cm), low risk, unifocal, intrathyroidal papillary carcinomas in the absence of prior head and neck irradiation or radiologically or clinically involved cervical nodal metastases (cN1a)	Lobectomy	A	–
	Clinically involved central or lateral neck lymph nodes (cN1)	Therapeutic CLND	B	56, 139, 181
	Advanced primary tumors (T3 or T4)	Prophylactic CLND	C	174, 175
	Small (T1 or T2), noninvasive, clinically node-negative PTCs and most FTC	Near-total or total thyroidectomy without prophylactic CLND	C	–
	Biopsy-proven metastatic lateral cervical lymphadenopathy.	Therapeutic LLND	B	–
2012 Chinese guidelines	MTD > 4 cm, or with gross extrathyroidal extension (cT4), or cN1, or distant sites (cM1)	Near-total or total thyroidectomy	C	46
	MTD > 1 cm and <4 cm without extrathyroidal extension (ETE), and cN0	Near-total or total thyroidectomy or lobectomy	C	45
	MTD < 1 cm without ETE and cN0 in the absence of prior head and neck irradiation, and no nodules in the contralateral lobe	Lobectomy	C	–
	Differentiated thyroid cancer	CLND	B	47, 48, 49
	Biopsy-proven metastatic lateral cervical lymphadenopathy	Therapeutic LLND	B	52, 53
2015 ATA guidelines	MTD > 4 cm, or with cT4, or cN1, or distant sites (cM1)	Near-total or total thyroidectomy	Strong	318, 319, 320, 321
	MTD > 1 cm and <4 cm without ETE, and cN0	Near-total or total thyroidectomy or lobectomy	Strong	318, 322, 323, 324, 325, 326, 327
	MTD < 1 cm without ETE and cN0 (unless there are clear indications to remove the contralateral lobe)	Lobectomy	Strong	–
	cN1a	Therapeutic CLND	Strong	334, 346, 347, 349, 359–366
	PTC with cN0 who have advanced primary tumors (T3 or T4), cN1b, or if the information will be used to plan further steps in therapy	Prophylactic CLND	Weak	344, 345, 353–356
	Small (T1 or T2), noninvasive, clinically node-negative PTC and for most follicular cancers	Thyroidectomy without prophylactic CLND	Strong	–
	Biopsy-proven metastatic lateral cervical lymphadenopathy	Therapeutic LLND	Strong	379–381

^a^References are numbered as in the original guideline

## Materials and methods

### Study design

A retrospective longitudinal study was conducted in line with STROCSS criteria [25].

### Setting

The Thyroid Surgery Division in China–Japan Union Hospital of Jilin University is the largest referral and academic thyroid surgery center in the northeastern region of China. The number of outpatients exceeds 100,000 per year, and the number of inpatients exceeds 3500 per year. Patients are mainly from three northeastern provinces in China. The division is a training center for endocrine surgery in China with international cooperation with surgeons from Europe and Asia.

### Participants

All TC patients in the period between 2008 and 2017 who underwent thyroidectomy in the hospital were included in our study. Surgeries were performed by general surgeons with special training in TC surgery. TC diagnosis was based on histopathology reports from surgical specimens [26]. The exclusion criteria were as follows: occult cancer, patients who had surgery outside our hospital, recurrent TC or history of TC, incomplete follow-up, TC and parathyroid disease. Preoperative thyroid stimulating hormone, free thyroxine-4, and free thyroxine-3 measurements and neck ultrasonography were performed for all patients who qualified for surgical treatment [26]. Fine-needle aspiration biopsy (FNAB) and preoperative computer tomography were not routinely employed. All patients required L-tyrosine supplementation after surgical treatment [26]. The follow‐up period was from the date of surgery to the date of the last clinical follow‐up.

### Data source, collection, and variables

The Institutional Electronic Database of the Thyroid Surgery Division in China–Japan Union Hospital of Jilin University was queried to audit time trend patterns in TC management between 2008 and 2017. Information on patients treated in the unit is prospectively collected for continuous monitoring of quality indicators. Data include deidentified patient information on histology, preoperative work-up, surgery, multidisciplinary management, and follow-up. The local database was reviewed to identify patients who underwent surgery for primary TC. Data were collected through completion of a three-part patient data sheet. The first part included demographic data. The second part included information regarding the operation, such as the type of surgery, year of operation, and administration of RAI. The third part included information regarding the histopathological nature of the tumors. The institutional database represents 100% of the admissions in the Thyroid Surgery Division. Data can be weighted such that the results can be extrapolated. Procedure and diagnostic codes are recorded using the International Classification of Diseases, Clinical Modification.

### Surgery

Patients in this study underwent open or endoscopic surgeries. The primary lesion was excised at the same time that the cervical lymph nodes were dissected. According to the results of intraoperative frozen sections, ipsilateral lobe and isthmus resection was performed for unilateral primary lesions, total thyroidectomy (TT) was performed for unilateral primary lesions requiring iodine 131 treatment. We generally performed the lymph node dissection (LND) of ipsilateral cervical central VI, which was recommended in China [23]. For all bilateral primary lesions, TT and bilateral cervical central VI LND were performed. Additional lateral compartment neck dissections, including levels II–IV, were performed if metastases were present in the lateral compartment, and no level I or V LND was performed unless lymph node metastasis to level I or V.

### Definitions

Tumor stages were defined according to the American Joint Committee on Cancer Manual, 8th Edition [27]. The risk of recurrence was defined according to the risk stratification system of differentiated thyroid cancer (DTC) of American Thyroid Association (ATA) management guidelines published in 2015 [26].

### Outcomes analysis

The measured outcomes were newly treated TC, demographic data, pathological characteristics [maximum tumor diameter (MTD), location, multifocality, extrathyroid extension (ETE) (identified as “the invasion of the cancer penetrated through the capsule and even reached the outside of the thyroid gland: perithyroidal soft tissues or strap muscles”), thyroiditis, coexisting nodular goiter, histological subtype, lymph node metastasis (LNM)] and treatment strategies [thyroidectomy, lobectomy, LNDs, and RAI].

### Stratification

To obtain the time trend study population, TC patients were stratified into subgroups for subsequent analyses.

#### Age

An age-stratified analysis was performed for the following groups: children (<18 years); youth (19–44 years); middle age (45–59 years); elderly (60+ years).

#### Thyroid gland surgery

The patients were stratified by the extent of the primary surgical procedure: TT; near TT; sub-TT; lobectomy + isthmusectomy; <lobectomy (i.e., nodulectomy).

#### Lymph node dissection

Solely thyroidectomy (without LND); central LND (CLND); CLND + lateral LND (LLND); and LLND without CLND.

The decision to perform a different type of surgery was made jointly by the patient and surgeon. Technical aspects of the procedures have been described previously [26, 28].

#### TC guidelines

Temporal trends were evaluated over three time intervals to evaluate the distribution of surgical methods in each period, depending on the published time management guidelines for adult patients with TC [20–24, 26, 29]. Period I, before 2012 Chinese guidelines published (i.e., 2008–2012); Period II, after 2012 Chinese guidelines published (i.e., 2013–2015); and Period III, after 2015 ATA guidelines published (i.e., 2016–2017).

#### Pathology

Group I included patients with DTC < 1 cm, without ETE, without clinical evidence of any LNM (N0). Group II included patients with DTC > 1 cm and <4 cm or with microscopic ETE and N0. Group III included patients with DTC > 4 cm or with gross ETE (T4), multifocality, or clinically apparent metastatic disease to nodes (N1) or distant sites (M1). Papillary thyroid microcarcinoma (PTMC) had MTD < 1 cm.

#### Dominant vs. contralateral thyroid lobe features

To analyze possible causes of changes in surgical management during the study period, we evaluated the US characteristics of the dominant and contralateral thyroid nodules. By definition, the dominant lobe is the larger thyroid lobe, the lobe affected by a suspected or proven tumor, the side with a hyperfunctioning hot nodule, and in a multinodular goiter, the lobe with larger and/or the greater number of nodules or the lobe causing compression symptoms [30]. Thyroid nodules were defined according to thyroid imaging reporting and data system (TI-RADS) classification [20, 31–33].

### Bias

Selection bias was reduced by including all the TC patients. In addition, given to the observational design of this study, the indication bias could not be excluded.

### Statistical analysis

Continuous variables were summarized by arithmetic means, standard deviations, and medians, and categorical values by weighted frequency and percentage (%). Data were analyzed using Student’s *t* test and Kruskal–Wallis *H* test for continuous variables and chi-square test for categorical variables. A *p* value < 0.05 was considered statistically significant. All hypothesis tests were two sided. All the data were analyzed retrospectively by a statistician. Statistical analyses were performed using IBM SPSS version 19.0, and charts were generated using Origin version 8.0.

## Results

### Population

**Fig. 1:**
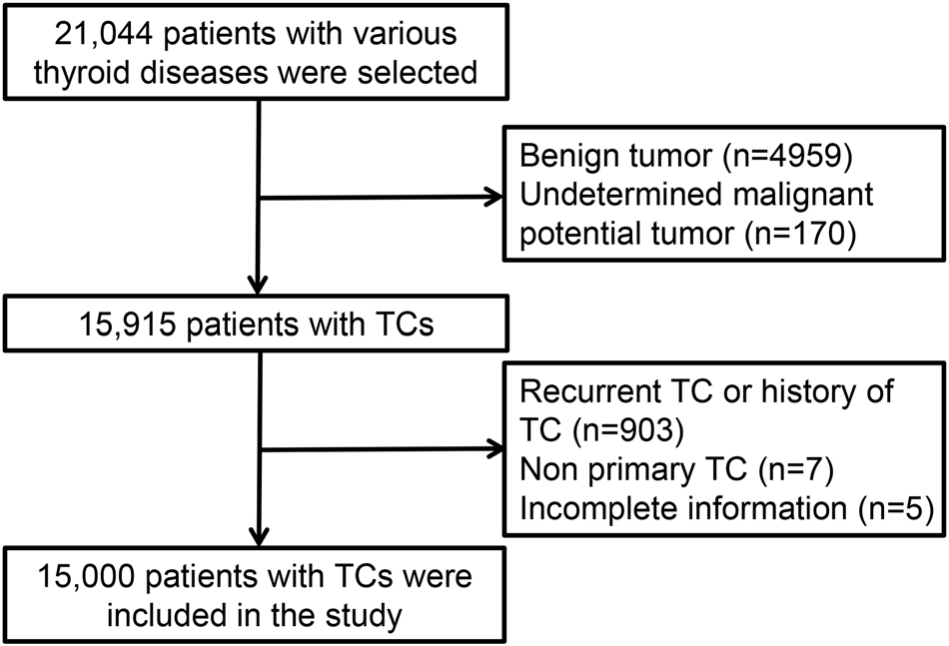
Flowchart of the inclusion and exclusion criteria. TC thyroid cancer

During the 10-year study period, 21,044 patients underwent surgery for various thyroid diseases. A total of 15,915 (75.6%) patients had a diagnosis of TC, and 915 (5.7%) were excluded from the analysis. Among them, 903 (5.6%) subjects underwent reoperation, 5 (0.03%) had incomplete information, and 7 (0.04%) had nonprimary TC. Finally, the analysis included 15,000 patients who underwent primary surgery with TCs and complete information for this study (Fig. 1).

### Surgical volume

Of the 15,000 TC patients selected, 214 (1.4%) underwent endoscopic surgery, and previous studies published by the center have compared the safety of endoscopic surgery and open surgery [34]. Preoperative FNA use increased from 0 to 92% over the 10-year period (Fig. S1).

### Demographics

A total of 11,805 patients were females (78.7%), with a male to female ratio of 1:3.69. There were significant differences in the sex ratio during the study period. The mean age was 42.88 ± 9.56 years. TC was most common in female youth (19–44 years). The highest TC incidence was observed in youth (56.5%), followed by the middle-aged group (39%). Elderly patients and children constituted 4% and 0.3% of the total, respectively. During the 10-year period, the age group distribution was significantly different (*p* < 0.05) (Table 2).

**Table 2:** The distribution of the clinical-pathological characteristics of thyroid cancer, *n* (%)

Year of diagnosis	Total	2008	2009	2010	2011	2012	2013	2014	2015	2016	2017	*P* value
Total	15,000	151	574	691	847	1130	1770	2233	2265	2482	2857	
Sex												
Male	3195 (21.3)	27 (17.9)	102 (17.8)	142 (20.6)	185 (21.8)	246 (21.8)	430 (24.3)	482 (21.6)	484 (21.4)	502 (20.2)	595 (20.8)	0.043
Female	11,805 (78.7)	124 (82.1)	472 (82.2)	549 (79.4)	662 (78.2)	884 (78.2)	1340 (75.7)	1751 (78.4)	1781 (78.6)	1980 (79.8)	2262 (79.2)	
Age groups												
Children (<18 years)	40 (0.3)	0 (0)	0 (0)	3 (0.4)	3 (0.4)	5 (0.4)	4 (0.2)	5 (0.2)	9 (0.4)	2 (0.1)	9 (0.3)	<0.05
Youth (19–44 years)	8475 (56.5)	86 (57.0)	331 (57.7)	390 (56.4)	497 (58.7)	635 (56.2)	1018 (57.5)	1334 (59.7)	1298 (57.3)	1394 (56.2)	1492 (52.2)	
Middle age (45–59 years)	5864 (39.1)	57 (37.8)	223 (38.9)	267 (38.6)	324 (38.3)	434 (38.4)	693 (39.2)	823 (36.9)	851 (37.6)	979 (39.4)	1213 (42.5)	
Elderly (60+ years)	621 (4.1)	8 (5.3)	20 (3.5)	31 (4.5)	23 (2.7)	56 (5.0)	55 (3.1)	71 (3.2)	107 (4.7)	107 (4.3)	143 (5.0)	
Histological type												
PTC	14,916 (99.4)	146 (96.7)	567 (98.8)	684 (99.0)	842 (99.4)	1122 (99.3)	1763 (99.6)	2222 (99.5)	2252 (99.4)	2473 (99.6)	2845 (99.6)	>0.05
FTC	6 (0.04)	2 (1.3)	1 (0.2)	0 (0)	0 (0)	0 (0)	0 (0)	0 (0)	0 (0)	1 (0.1)	2 (0.1)	
MTC	73 (0.5)	1 (0.7)	6 (1.1)	7 (1.0)	5 (0.6)	8 (0.7)	7 (0.4)	9 (0.4)	12 (0.5)	8 (0.3)	10 (0.4)	
ATC	5 (0.03)	2 (1.3)	0 (0)	0 (0)	0 (0)	0 (0)	0 (0)	2 (0.09)	1 (0.1)	0 (0)	0 (0)	
MTD (median, cm)	0.6	–	0.8	0.7	0.6	0.6	0.6	0.6	0.6	0.6	0.6	>0.05
Multifocality	6052 (40.4)	40 (26.5)	184 (32.1)	272 (39.4)	325 (38.4)	425 (37.6)	700 (39.5)	963 (43.1)	947 (41.8)	1051 (42.3)	1145 (40.1)	<0.05
Extrathyroid extension	2018 (13.5)	4 (2.7)	13 (2.3)	41 (5.9)	45 (5.3)	102 (9.0)	221 (12.5)	236 (10.6)	375 (16.6)	427 (17.2)	554 (19.4)	<0.05
Nodular goiter present	11,642 (77.6)	126 (83.4)	491 (85.5)	406 (58.8)	620 (73.2)	913 (80.8)	1435 (81.1)	1777 (79.6)	1664 (73.5)	1822 (73.4)	2388 (83.6)	>0.05
Thyroiditis present	3285 (21.9)	48 (31.8)	174 (30.3)	201 (29.1)	235 (27.7)	255 (22.6)	358 (20.2)	402 (18)	497 (21.9)	481 (19.4)	634 (22.2)	>0.05
LNM	6326 (42.2)	44 (29.1)	198 (34.5)	299 (43.3)	317 (37.4)	404 (35.8)	738 (41.7)	968 (43.4)	1039 (45.9)	1095 (44.1)	1224 (42.8)	<0.05
Central LNM	3706 (24.7)	13 (8.6)	96 (16.7)	118 (17.1)	104 (12.3)	126 (11.2)	340 (19.2)	567 (25.4)	622 (27.5)	790 (31.8)	930 (32.6)	
Lateral LNM	2620 (17.5)	31 (20.5)	102 (17.8)	181 (26.2)	213 (25.1)	278 (24.6)	398 (22.5)	401 (18)	417 (18.4)	305 (12.3)	294 (10.3)	
Lobes of cancer												
Left lobe	5012 (33.4)	63 (41.7)	199 (34.7)	218 (31.6)	278 (32.8)	379 (33.5)	619 (35.0)	727 (32.6)	761 (33.6)	818 (33.0)	950 (33.3)	<0.05
Right lobe	5731 (38.2)	59 (39.1)	238 (41.5)	261 (37.8)	309 (36.5)	465 (41.2)	652 (36.8)	846 (37.9)	820 (36.2)	950 (38.3)	1131 (39.6)	
Isthmus	153 (1.0)	6 (4.0)	18 (3.1)	17 (2.5)	26 (3.1)	15 (1.3)	15 (0.9)	16 (0.7)	13 (0.6)	12 (0.5)	15 (0.5)	
Bilaterlity	4100 (27.3)	23 (15.2)	119 (20.7)	195 (28.2)	232 (27.4)	271 (24.0)	484 (27.3)	644 (28.8)	670 (29.6)	702 (28.3)	760 (26.6)	
8th AJCC/TNM stage												
Stage I	14,383 (95.9)	134 (88.7)	545 (95.0)	667 (96.5)	825 (97.4)	1089 (96.4)	1715 (96.9)	2139 (95.8)	2165 (95.6)	2366 (95.3)	2738 (95.8)	<0.05
Stage II	531 (3.5)	1 (0.7)	19 (3.3)	19 (2.8)	18 (2.1)	34 (3.0)	52 (2.9)	87 (3.9)	89 (3.9)	105 (4.2)	107 (3.8)	
Stage III	23 (0.2)	2 (1.3)	1 (0.2)	2 (0.3)	0 (0)	2 (0.2)	1 (0.1)	3 (0.1)	4 (0.2)	5 (0.2)	3 (0.1)	
Stage IV	41 (0.3)	2 (1.3)	1 (0.2)	3 (0.4)	2 (0.2)	5 (0.4)	2 (0.1)	4 (0.2)	7 (0.3)	6 (0.2)	9 (0.3)	
Unknown	22 (0.2)	12 (8.0)	8 (1.4)	0 (0)	2 (0.2)	0 (0)	0 (0)	0 (0)	0 (0)	0 (0)	0 (0)	
Risk stratification of recurrence												
Low risk	8761 (58.9)	58 (39.2)	380 (66.9)	389 (57.1)	498 (59.4)	683 (61.2)	1027 (58.4)	1379 (62.2)	1295 (57.7)	1427 (57.7)	1625 (57.3)	<0.05
Intermediate risk	5683 (38.2)	86 (58.1)	175 (30.8)	268 (39.4)	319 (38.0)	403 (36.1)	687 (39.1)	778 (35.1)	876 (39.1)	967 (39.1)	1124 (39.6)	
High risk	438 (2.9)	4 (2.7)	13 (2.3)	24 (3.5)	22 (2.6)	31 (2.8)	45 (2.6)	60 (2.7)	72 (3.2)	78 (3.2)	89 (3.1)	

### Histopathology

Of 15,000 TCs, 14,916 were PTCs (99.4%), 73 were medullary (0.5%), 6 were follicular (0.04%), and 5 were anaplastic (0.03%) (Table 2). The median MTD was 0.6 (0.01–6.5) cm. Coexistence of goiter or thyroiditis occurred in 11,642 (77.6%) or 3285 (21.9%) histological specimens, severally, and remained stable throughout the study period. Incidence of ETE increased from 2.6 to 19.3% over time. TC multifocality increased from 26.4 to 40.1%. LNM incidence increased from 29.2 to 42.8%, which mainly depended on the increased incidence of central LNM (CLNM), while the rate of lateral LNM was increased from 20.5% in 2008 to 26.2% in 2010 and decreased to 10.3% until 2017. The most common location of TC was the right side (*n* = 5731, 38.2%), followed by the left lobe (*n* = 5012, 33.4%), bilaterally (*n* = 4100, 27.3%), and the isthmus (*n* = 153, 1.0%). Group I (i.e., DTC < 1 cm, without ETE, N0) included 4700 patients (31.5%); Group II (DTC > 1 and <4 cm, or with microscopic ETE, N0) included 830 patients (5.5%); and Group III (DTC > 4 cm, or with gross ETE, N1, M1) included 9391 patients (62.9%). Stratification features remained stable during the study period. Stage I and low-risk patients remained prevalent during the 10-year period (95.9% and 58.9%, respectively) (Fig. S2). Of the patients, 13,897 were pT1 (92.6%), 8674 (53.8%) were pN0, and 14,964 (99.7%) were M0. PTMC was found in 11,403 patients (76.5%) and non-PTMC in 3513 patients (23.6%). The number of PTMC increased over time. Among all the patients undergoing surgery, only six patients had positive margins.

### Management methods

#### Trends in the extent of thyroidectomy

**Fig. 2:**
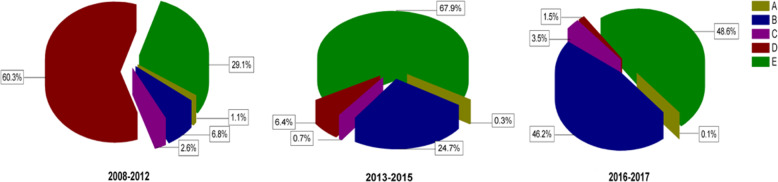
The distribution of thyroidectomies for thyroid cancer (TC). Percentages of thyroidectomies in three periods according to the time of published guidelines, where A was defined as <lobectomy, B was defined as lobectomy + isthmusectomy, C was defined as subtotal thyroidectomy, D was defined as near-total thyroidectomy, and E was defined as total thyroidectomy (TT)

**Fig. 3:**
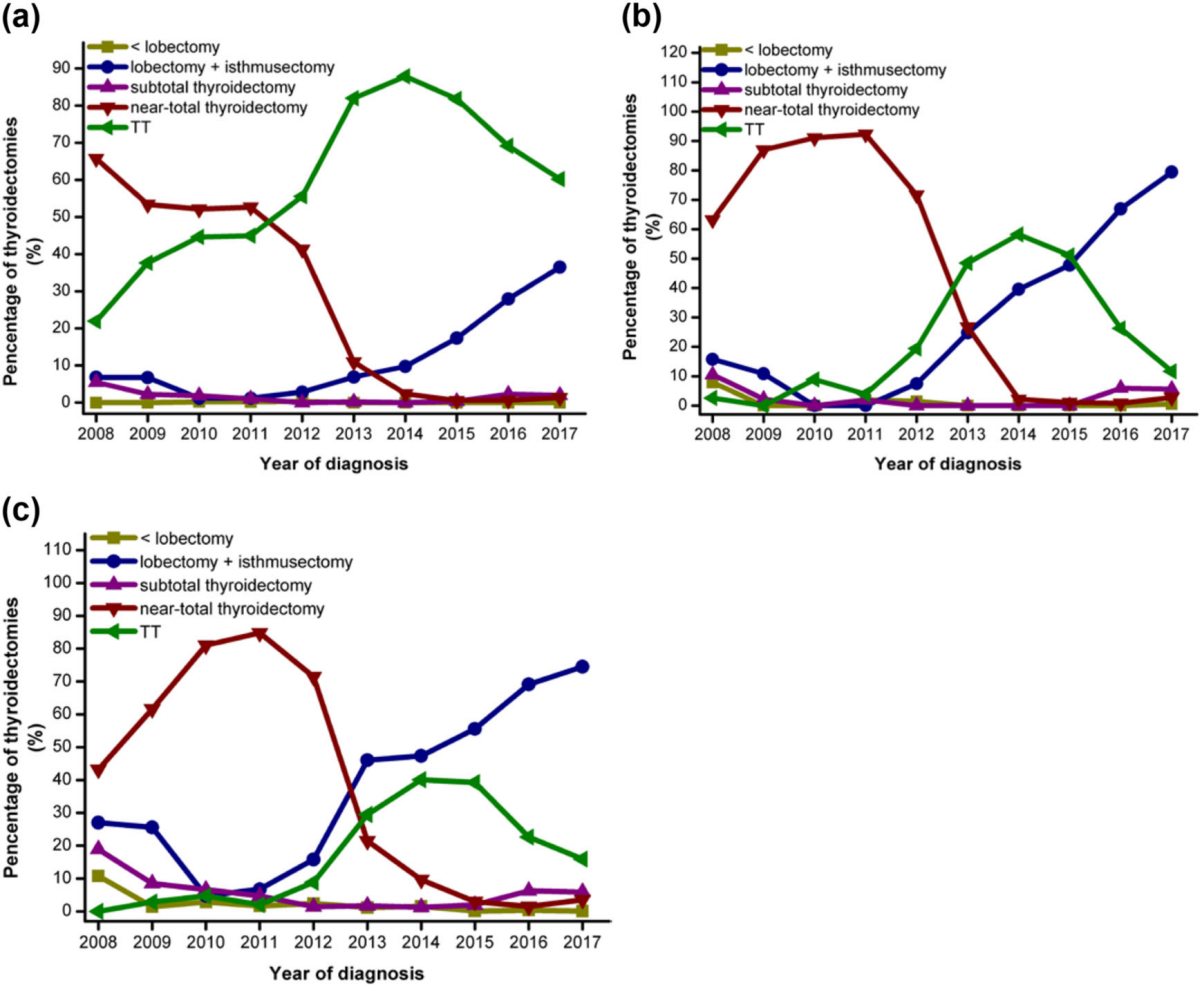
The distribution of thyroidectomies for differentiated thyroid cancer (DTC). Trends of percentages of thyroidectomies over the 10-year period for patients in Group III (**a**), in Group II (**b**), and in Group I (**c**). TT total thyroidectomy

In total, most patients underwent TT (*n* = 7844, 52.3%), followed by lobectomy + isthmusectomy (*n* = 4249, 28.3%), near TT (*n* = 2527, 16.8%), sub-TT (*n* = 320, 2%), and nodulectomy (*n* = 64, 0.4%). Before 2012 Chinese guidelines published (from 2008 to 2012), the most common surgery was near TT (*n* = 2045, 60.3%), followed by TT (*n* = 989, 29.1%), lobectomy + isthmusectomy (*n* = 232, 6.8%), and sub-TT (*n* = 90, 2.6%). After 2012 Chinese guidelines published (from 2013 to 2015), the rate of near TT decreased to 6.4%, while the rate of TT increased to 67.9%, and the rate of lobectomy + isthmusectomy increased to 24.7%. Since 2015 ATA guidelines published (from 2016 to 2017), the rate of near TT decreased to 1.5%, the rate of TT decreased to 48.6%, and the rate of lobectomy + isthmusectomy increased to 46.2%. (Fig. 2). To evaluate the distribution thyroidectomies for DTC over the 10-year period, we divided 15,000 patients into three groups by tumor features, and noted that the majority of patients in Groups I and II received lobectomy + isthmusectomy (48.3% and 41.3%, respectively), and the majority of patients in Group III underwent TT (69.0%). The tendencies for thyroidectomies in three Groups were shown in Fig. 3.

#### Dominant vs. contralateral thyroid lobe features

In order to analyze the reasons why the patients with similar pathology features chose different surgery than those guidelines recommended, we evaluated thyroid lobe features in these three groups. In Group I patients who received near TT or TT, the TI-RADS classification was higher than grade IV in the contralateral thyroid nodules (37%), followed by multiple nodules > 1 cm (32%), nodules > 3 cm (21%), patient symptoms (8%), and false-positive FNA result (2%). In Group II, the patients who underwent lobectomy + isthmusectomy included no nodule (59%), nodule < 8 mm and TI-RADS < grade III (24%) in the contralateral lobe, negative FNA result (13%), and patient symptoms (4%). In Group III, patients who received lobectomy + isthmusectomy included negative FNAB results (63%), no nodule (33%), nodule < 8 mm and TI-RADS < grade III (12%) in the contralateral lobe, and patient symptoms (2%) (Table 3).

**Table 3:** Possible reasons for the choice of surgeries among the three groups

	Patients^a^ in Group I	Patients^b^ in Group II	Patients^c^ in Group II	Patients^d^ in Group III
False-positive result of FNAB	2%	1%	–	–
Nodules > 3 cm	21%	17%	–	–
Multiple nodules > 1 cm	32%	23%	–	–
TI-RADS classification > grade IV	37%	54%	–	–
Negative result of FNAB	–	–	13%	63%
No nodule	–	–	59%	33%
Nodule < 8 mm with TI-RADS classification < grade III	–	–	24%	12%
Patient symptoms	8%	5%	4%	2%

*TI-RADS* thyroid imaging reporting and data system

^a^Patients who underwent near or total thyroidectomy in Group I

^b^Patients who underwent near or total thyroidectomy in Group II

^c^Patients who underwent lobectomy + isthmusectomy in Group II

^d^Patients who underwent lobectomy + isthmusectomy in Group III

#### Trends in LND

**Fig. 4:**
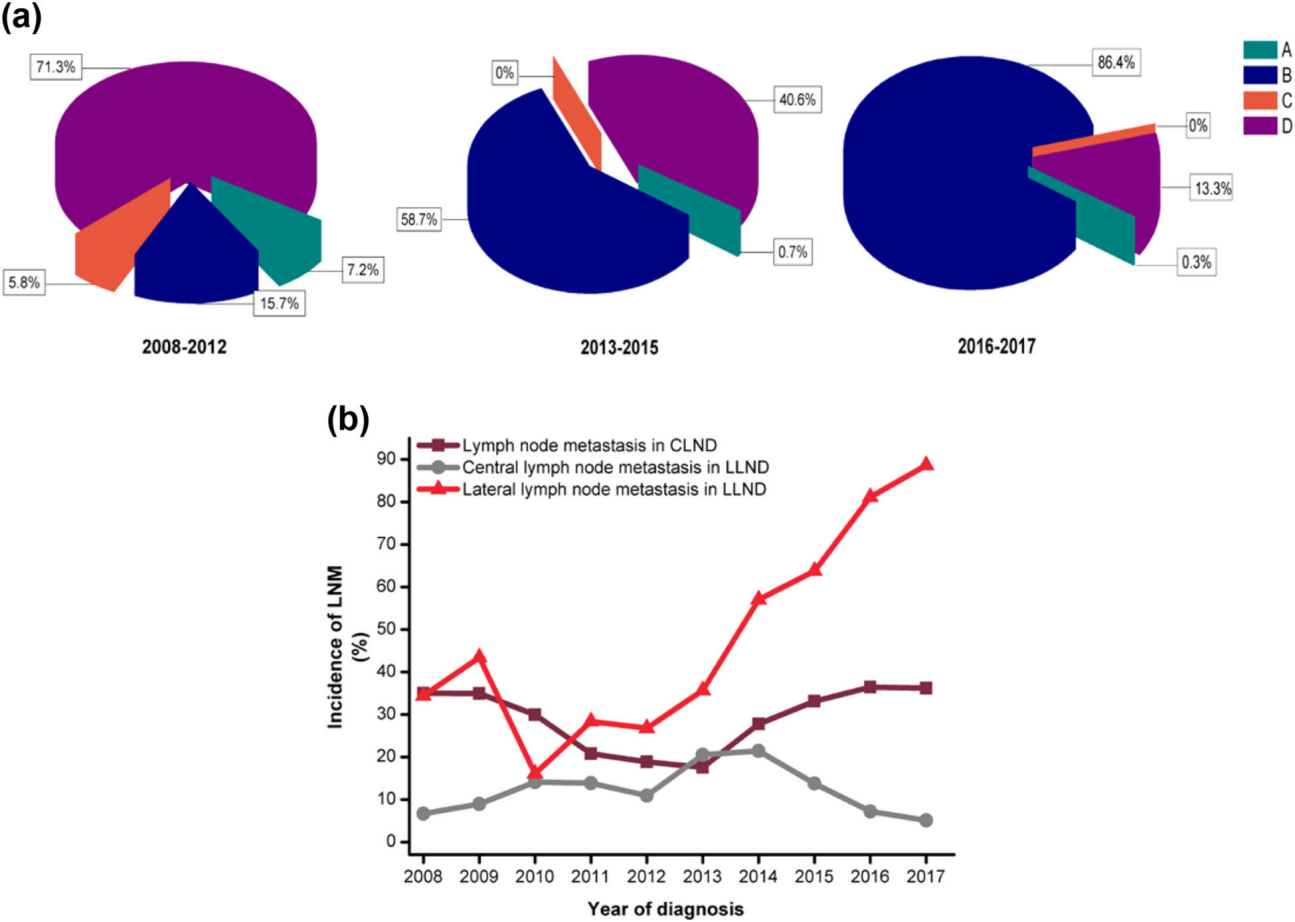
The distribution of lymph node dissections (LNDs). **a** Percentages of LNDs in the three groups according to the period of published guidelines, where A was defined as underwent thyroidectomy without LND, B was defined as central LND (CLND), C was defined as only lateral LND (LLND) without CLND, and D was defined as CLND + LLND. **b** The incidence of lymph node metastasis (LNM) in patients who underwent LNDs and the incidence of LNM were divided by the number of patients who underwent LNDs (CLND and CLND + LLND) by the number of patients with LNM (central LNM and lateral LNM)

A total of 8827 patients (58.8%) underwent CLND, and 5675 patients (37.8%) underwent CLND + LLND. Three hundred patients (2%) underwent thyroidectomy without LND. A total of 198 patients (1.3%) underwent LLND without CLND. Before 2012 Chinese guidelines published (from 2008 to 2012), the most common surgery was CLND + LLND (*n* = 2420, 71.3%), followed by CLND (*n* = 532, 15.7%), thyroidectomy without LND (*n* = 243, 7.2%), and LLND without CLND (*n* = 198, 5.8%). After 2012 Chinese guidelines published (from 2013 to 2015), the rate of CLND increased to 58.7%, while the rate of CLND + LLND decreased to 40.6%, the rate of thyroidectomy without LND and LLND without CLND had dropped to close to 0. Since 2015 ATA guidelines published (from 2016 to 2017), the rate of CLND increased to 86.4%, however, the rate of CLND + LLND decreased to 13.3% (Fig. 4a). We then evaluated the accuracy of LNDs. CLNM in patients undergoing central compartment clearance decreased from 35% in 2008 to 17.5% in 2013 and then increased to 36.2% in 2017 (Fig. 4b). CLNM (N1a) of patients undergoing CLND + LLND increased from 6.6% in 2008 to 21.4% in 2014 and decreased to 5% in 2017. Lateral LNM (N1b) of patients undergoing CLND + LLND increased from 34.4% in 2008 to 88.6% in 2017.

#### RAI

**Fig. 5:**
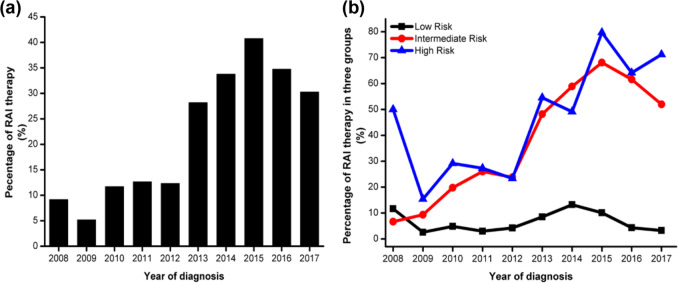
The distribution of patients undergoing radioactive iodine (RAI) therapy. **a** Trends in the percentage of RAI therapy over the 10-year period, and the percentage was divided by the number of adult (18+ years) patients with differentiated thyroid cancer (DTC) who underwent near-total or total thyroidectomy by the number of patients undergoing RAI therapy. **b** Percentages of RAI therapy in three risk groups according to the 2015 American Thyroid Association (ATA) guidelines. The percentage was divided by the number of adult patients with DTC who underwent near-total or total thyroidectomy by the number of patients undergoing RAI therapy in three groups

Of the 10,273 adults (>18 years) who underwent near TT or TT, 2870 (27.6%) patients received RAI. The RAI therapy feature showed a stable trend in 2008–2012 (10%), except for a low value in 2009 (5.4%), which increased from 12.3% in 2012 to 41.3% in 2015 and decreased to 32.4% in 2017 (Fig. 5a). In total, 5473 (51.6%) were low-risk patients, 4681 (44.8%) were intermediate-risk patients, and 394 (3.7%) were high-risk patients. Low-risk and intermediate-risk patients increased from 2008 to 2015 (Table 2). Trends of the percentage of patients undergoing RAI therapy in different risk groups were shown in Fig. 5b. The percentage of RAI therapy in the low-risk group was <5%. RAI therapy in the intermediate-risk group increased from 6.6% in 2008 to 26% in 2011, remained stable in 2011–2012, increased to 68% in 2015, and decreased to 52.0% in 2017. RAI therapy in the high-risk group showed an overall increasing trend during the period 2008–2015 and decreased until 2017 (71.2%); however, there was a plateau period from 2010 to 2012, and the percentage in the years 2009 and 2014 separately was lower than that in the previous year.

## Discussion

### What was the impact of guideline statements endorsing TT or lobectomy for TC on final surgical treatment during the last 10-year period?

Endocrine surgeons are increasingly aware of the need to address overtreatment in TC care [20–24, 33]. TC exemplifies these concerns because most newly diagnosed patients with a favorable prognosis are treated with multiple modalities for which the benefit of each treatment may be small, but the burden is cumulative and substantial [19]. This survey was designed to collect information on TC surgical management and present a 10-year trend. In a sample of 15,000 TC patients undergoing thyroid surgery between 2008 and 2017, the most notable finding was the decreasing proportion of patients receiving TT (from 71 to 41%) and the increasing proportion receiving lobectomy—from 14 to 50%. However, TC patients who underwent lobectomy also underwent LNDs significantly more frequently. Furthermore, in our institution, FNAB use increased from 0 to 90%. TT was the most common treatment during the early period, but the emergence of lobectomy has triggered a shift. The use of lobectomy has increased after years of steadily decreasing rates. The decrease in TT surgery after lobectomy demonstrates the benefit of evidence-based guidelines in accelerating changes to clinical practice to reduce overtreatment [20–24, 26]. Although lobectomy as a surgical approach has lower morbidity, an important downside to its use is the historically high rate of additional reoperations after initial lobectomy, ranging from 5 to 10% in published reports [35]. According to the data in our center, six patients (1.4%) underwent primary lobectomy and reoperation for recurrence. These data, however, did not fully represent the incidence of recurrence for patients with lobectomy. The authors found that prophylactic CLND can definitely reduce the recurrence rate of DTC [36, 37]. The increase in lobectomy was accompanied by a marked increase in the use of additional CLND after initial lobectomy during the study period. We also found that the prophylactic LLND was gradually giving a way to therapeutic LLND in the last decade. These variations were mainly because prophylactic LLND was not recommended, while prophylactic CLND has been recommended by the Chinese guidelines since 2012 [23]. In addition, the increased rate of lateral LNM may depend on preoperative information availability from FNAB. We also found that the treatment strategy in our institution was adjusted according to the features of the contralateral lobe of cancer foci.

### Clinicopathological trends

Our study showed that the most common histological type of TC was PTC. PTMC also increased over time. These might be associated with the increased rate of TC screening in China and use of FNA in our institution. Cho et al. also reached a similar conclusion that the incidence of TC might be associated with the increased use of US screening [38]. In the present study, we found that TCs were limited to early stage (stage I, and low risk) tumors. The prevalence of lateral LNM was 10.3% in 2017, lower than 20.9% in a meta-analysis [39]. These results suggest that patients with TC in northeast China were more likely to have a favorable prognosis. However, we thought that clinicians should pay more attention to these early stage PTCs, which also showed increasing rates of multifocality and ETE over time. TC was most common in youth (19–44 years). Vaccarella et al. compared the age-specific incidence rates of TC among different countries and found that TC increased, particularly in young/middle-aged individuals [40]. Similarly, Araque et al. reported an increasing incidence of TC between the ages of 15 and 39 in the United States [41]. In addition, it was interesting to found that the incidence of right lobe cancer was significantly higher than left lobe cancer. The specific reason for this result was still unclear and remains to further exploration.

### RAI therapy

In our study, we noted that the percentage of RAI therapy increased significantly during the period 2012–2015, and the number of intermediate- and high-risk patients and the percentage of RAI therapy in the above-mentioned two groups both increased rapidly. These data demonstrated that a substantial portion of the treated DTCs was overtreated during the period 2013–2015. The percentage of RAI therapy decreased from 2015 to 2017, consistent with the recommendations of the guidelines [26].

### Strengths and limitations

The strengths of the study include its very large, contemporary, diverse patient sample, as well as the collection of clinical and pathological information after the promulgation of the guidelines. However, there were some limitations. The incidence and mortality of TC were not analyzed in this study, and the pathology specimens were not reanalyzed. Follow-up and disease recurrence information are not included in the current data. In the future, relevant clinical studies will be conducted with a focus on these issues.

## Conclusions

We have demonstrated a significant decrease in the use of TT between 2008 and 2017, which resulted in a significant increase in the overall rate of lobectomy. This change seems to be associated with a change in the surgical approach regarding what constitutes adequate TC guideline adherence. Our findings also indicate that evidence-based multidisciplinary guidelines that address issues of clinical controversy can effectively accelerate changes in clinical practice and reduce overtreatment in TC care.

## Supplementary information

Supplementary Figure 1

Supplementary Figure 2
