# Supplementary figures and images for: Time trend analysis of thyroid cancer surgery in China: single institutional database analysis of 15,000 patients
Source: Endocrine. 2020 Mar 2;68(3):617–28. doi: 10.1007/s12020-020-02230-7 (PMC7308255; doi:10.1007/s12020-020-02230-7)

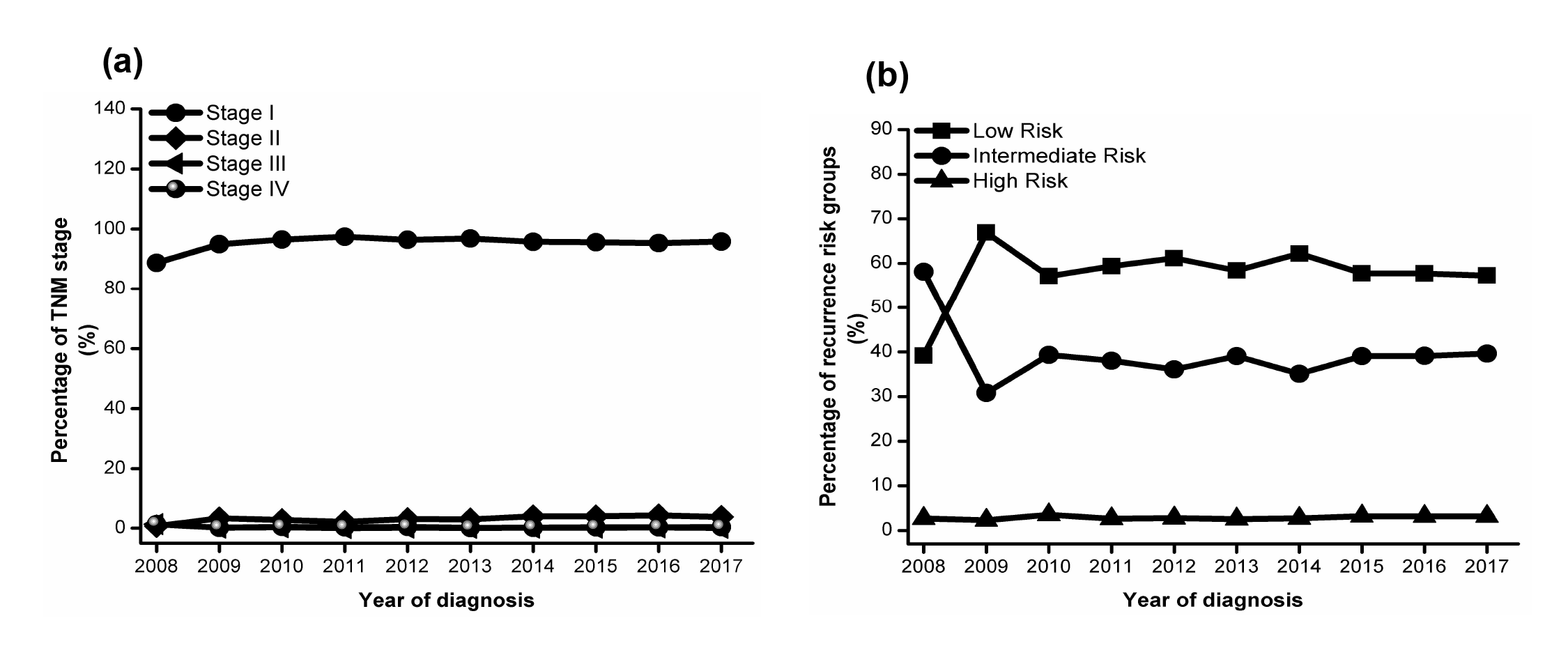

Supplement: Supplementary file 2 — Supplementary Figure 2 [file 12020_2020_2230_MOESM2_ESM.tif]
